# Increased mitochondrial and lipid metabolism is a conserved effect of Insulin/PI3K pathway downregulation in adipose tissue

**DOI:** 10.1038/s41598-020-60210-3

**Published:** 2020-02-25

**Authors:** Lucia Bettedi, Anqi Yan, Eugene Schuster, Nazif Alic, Lazaros C. Foukas

**Affiliations:** 1 Institute of Healthy Ageing and Department of Genetics, Evolution and Environment, University College London, London, WC1E 6BT UK; 2 Endocrinology Team, Breast Cancer Now, The Institute of Cancer Research, 237 Fulham Road, London, SW3 6JB UK; 3 Present Address: National Institutes of Child Health and Human Development (NICHD), Bethesda, MD 20814 USA

**Keywords:** Insulin signalling, Ageing

## Abstract

The Insulin/IGF-1 signalling (IIS) pathway plays an essential role in the regulation of glucose and lipid homeostasis. At the same time, a reduction in the IIS pathway activity can extend lifespan and healthspan in various model organisms. Amongst a number of body organs that sense and respond to insulin/IGF-1, the adipose tissue has a central role in both the metabolic and lifespan effects of IIS at the organismal level. Genetic inactivation of IIS components specifically in the adipose tissue has been shown before to improve metabolic profile and extend lifespan in various model organisms. We sought to identify conserved molecular mechanisms that may underlie the beneficial effects of IIS inhibition in the adipose tissue, specifically at the level of phosphoinositide 3-kinase (PI3K), a key IIS effector molecule. To this end, we inactivated PI3K by genetic means in the fly fat body and by pharmacological inhibition in mammalian adipocytes. Gene expression studies revealed changes to metabolism and upregulation of mitochondrial activity in mouse adipocytes and fly fat bodies with downregulated PI3K, which were confirmed by biochemical assays in mammalian adipocytes. These data suggest that PI3K inactivation has a conserved effect of upregulating mitochondrial metabolism in both fly and mammalian adipose tissue, which likely contributes to the health- and life-span extending effect of IIS pathway downregulation.

## Introduction

The fundamental mechanisms of cellular and organismal ageing remain an outstanding problem in biology. Nevertheless, intense research efforts of the past decades have greatly increased understanding of the ageing process. Notably, they have revealed genetic pathways that influence the rate of ageing. Well-studied genes that affect the rate of ageing, encode components of the insulin/IGF-1 signalling (IIS) pathway^1^. The IIS pathway plays an essential role in the regulation of metabolism at both cellular and whole body level. The interconnections between regulation of metabolism and rate of ageing, as exemplified in the case of IIS, has been an emerging theme in the recent years^2^.

The adipose tissue is the largest organ of the human body and it has been shown to be a master regulator of pathways involved in ageing, development of age-related dysfunctions and metabolic diseases^3,4^. This role of adipose tissue is evolutionarily conserved. Interventions specifically targeted at the adipose tissue have been shown to influence healthspan and lifespan in various experimental animals. Indeed, manipulations that affect lipid metabolism in the intestine, the equivalent of the adipose tissue, of the nematode worm *C. elegans* have been shown to affect the rate of ageing of this species^5^. In the fruitfly *D. melanogaster*, the fat body is the functional homolog of mammalian liver and adipose tissue^6^. Moreover, lipid metabolism in the adipocytes of the fat body, in terms of fat storage and mobilisation, is remarkably similar to that of mammalian white adipose tissue^7^. Genetic interventions targeted specifically at the IIS in the fat body have been shown to recapitulate the life-extending effects of the respective ubiquitous mutations. A notable example is overexpression of the transcription factor dFoxo in the fat body^8^. However, the underlying mechanisms of such beneficial effects remain elusive. In the case of loss-of-function mutations in components of IIS pathway, the ensuing activation of the transcription factor Foxo appears to be essential for the lifespan extending effect: in both *C. elegans* and *Drosophila,* lifespan extending effects of reduced IIS are critically depended on the activity of daf-16/Foxo^9,10^. FoxO proteins have been shown to exert protective effects in cells by upregulating genes conferring antioxidant capacity, but they also are important regulators of glucose and lipid metabolism^11^. In mice, there are a few examples of adipose-tissue specific mutations in components of the IIS pathway that exert a beneficial metabolic effect. A seminal example is the beneficial metabolic effects of fat-specific knock-out of the Insulin Receptor (IR) (FIRKO mice)^12^. Notably, FIRKO mice also showed extended lifespan^13,14^. Furthermore, downregulation of mTOR Complex 1 in the adipose tissue by aP2-Cre mediated deletion of Raptor resulted in leanness due to increased energy expenditure^15^.

The PI3K/Akt and the Ras/ERK are the main effector pathways in insulin signalling^16^. The class I PI3K p110α has previously been shown to be the principal mammalian PI3K isoform activated downstream the insulin receptor^17^. Partial inactivation of p110α by ubiquitous heterozygous mutation has been shown to confer improved metabolic profile in aged mice and to modestly extend lifespan^18^. Furthermore, adipose tissue-specific inactivation of p110α resulted in leaner mice due to increased energy expenditure^19^. Importantly, long-term pharmacological inhibition of p110α has previously been shown to protect mice and rhesus monkeys from diet-induced obesity, highlighting both the utility of PI3K inhibitors in metabolic studies and their therapeutic potential^20,21^. Although a worm ortholog of class I PI3K was the first gene ever reported to affect the rate of ageing^22^, the specific roles of PI3K in the context of processes associated with ageing have barely been investigated. The aim of the present study was to identify mechanisms underlying the effects of PI3K p110α inhibition at the adipocyte level and to establish their evolutionary conservation in the context of the previously reported beneficial effects of IIS pathway inhibition in the adipose tissue. To this end, we performed a comparative study aiming to reveal similarities and differences in gene expression and phenotypes ensuing from PI3K inactivation in fly fat body and mouse adipocytes. As a fly model, we used fat-body specific overexpression of a dominant negative form of the fly class I PI3K orthologue Dp110 (Dp110^DN^)^23^. As a mouse model, we employed 3T3-L1 adipocytes, the most widely used adipocyte model in conjunction with pharmacological inactivation of PI3K p110α exploiting the extensively characterised small molecule ATP competitive inhibitor A66^24^. We applied an unbiased transcriptome analysis in conjunction with biochemical assays in order to assess the effect of IIS downregulation, through PI3K inhibition, on adipocyte gene expression and function. We report here that inactivation of PI3K upregulates genes involved in mitochondrial activity and lipid metabolism in both fly fat body and mammalian adipocytes. This suggests that increased mitochondrial activity and lipid metabolism might be a conserved effect of PI3K inhibition, which contributes to lifespan extension through IIS downregulation.

## Results

### Downregulation of Dp110 signalling increases mitochondrial gene expression in fly fat body

**Figure 1:**
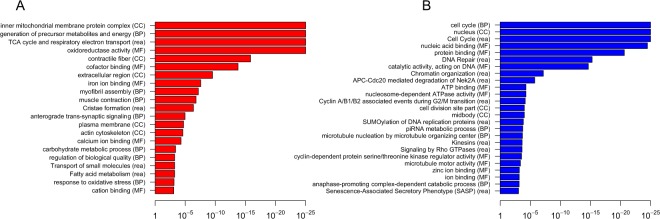
Gene expression alterations in fly fat body overexpressing Dp110^DN^. Enrichment of Gene Ontology (Biological Process-BP, Molecular Function-MF, Cellular Component-CC) and REACTOME (rea) annotation in significantly (adjusted P value <0.001) upregulated (**A**) and downregulated (**B**) genes in isolated fat bodies expressing Dp110^DN^ transgene compared to ‘driver-only’ (*FB-GAL4*) fat bodies. Enriched terms determined by gprofiler in R with strong hierarchical filtering strength.

In order to study the molecular effects of Dp110 inhibition in fly fat body, we applied an unbiased transcriptomic approach. We performed RNA-seq in fat bodies of flies overexpressing a dominant-negative Dp110 transgene (Dp110^DN^) under a fat body-specific driver (*FB-Dp110*^*DN*^ flies). Using this system, we detected an approximately 7-fold higher mRNA expression of the Dp110^DN^ transgene compared to that of the endogenous Dp110 gene (Supplementary Fig. 1). We found 723 genes differentially expressed by Dp110^DN^ overexpression (Supplementary Data 1). Genes associated with metabolism and mitochondrial function (respiratory chain) were the most prominent GO group upregulated in the *Dp110*^*DN*^ fly fat body (Fig. 1). On the other hand, the most prominent gene categories downregulated in Dp110^DN^ overexpressing fat bodies were genes involved in cell cycle regulation and genes related to antimicrobial defence. Importantly, the present findings are corroborated by a recent study that reported changes at the level of protein expression resulting from IIS pathway attenuation in flies lacking 3 out of 7 insulin-like peptides (DILPs)^25^. That study reported increased expression of mitochondrial electron transport chain proteins and demonstrated increased mitochondrial respiration in the fat body of mutant flies.

### Inhibition of the PI3K p110α signalling pathway regulates mitochondrial gene expression and increases mitochondrial respiration in mammalian adipocytes

**Figure 2:**
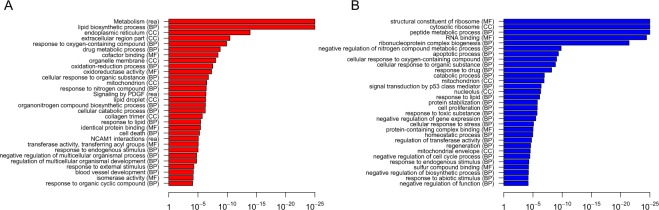
Gene expression alterations upon inhibition of PI3K p110α in 3T3-L1 adipocytes. Enrichment of Gene Ontology (Biological Process-BP, Molecular Function-MF, Cellular Component-CC) and REACTOME (rea) annotation in significantly (adjusted p value <0.0001) upregulated (**A**) and downregulated (**B**) genes after 3T3-L1 adipocytes were treated with the p110α-selective inhibitor A66 for 24 h. Enriched terms determined by gprofiler in R with strong hierarchical filtering strength.

To investigate the effect of p110α inactivation on mammalian adipocytes and to delineate an analogy with the trascriptome profile of the fly model describe above, we treated mouse 3T3-L1 adipocytes with A66, an extensively characterised and widely used p110α-selective inhibitor^24^, for 24 h, followed by RNA extraction. Gene expression analysis by RNA-seq (Supplementary Data 2) showed increased expression of genes associated with lipid metabolism and the mitochondria (Fig. 2). Downregulated genes were associated with ribosomal proteins and the regulation of protein translation. Therefore, the upregulation of mitochondrial genes by PI3K inhibition was conserved between flies and mice. However, we found a limited number of upregulated orthologous genes in both the *Dp110*^*DN*^ fly fat body and A66-treated 3T3-L1 adipocytes (including Aco2, Aldoa, Cpm, Acss2, Pcyt2 and the encoded NADH dehydrogenase 5) and only one orthologous gene downregulated in both conditions (FSCN1). This appears consistent with other studies looking at determinants of healthspan and ageing, where there was little evolutionary conservation at the individual gene level, but high conservation at the process and pathway level^26^.

**Figure 3:**
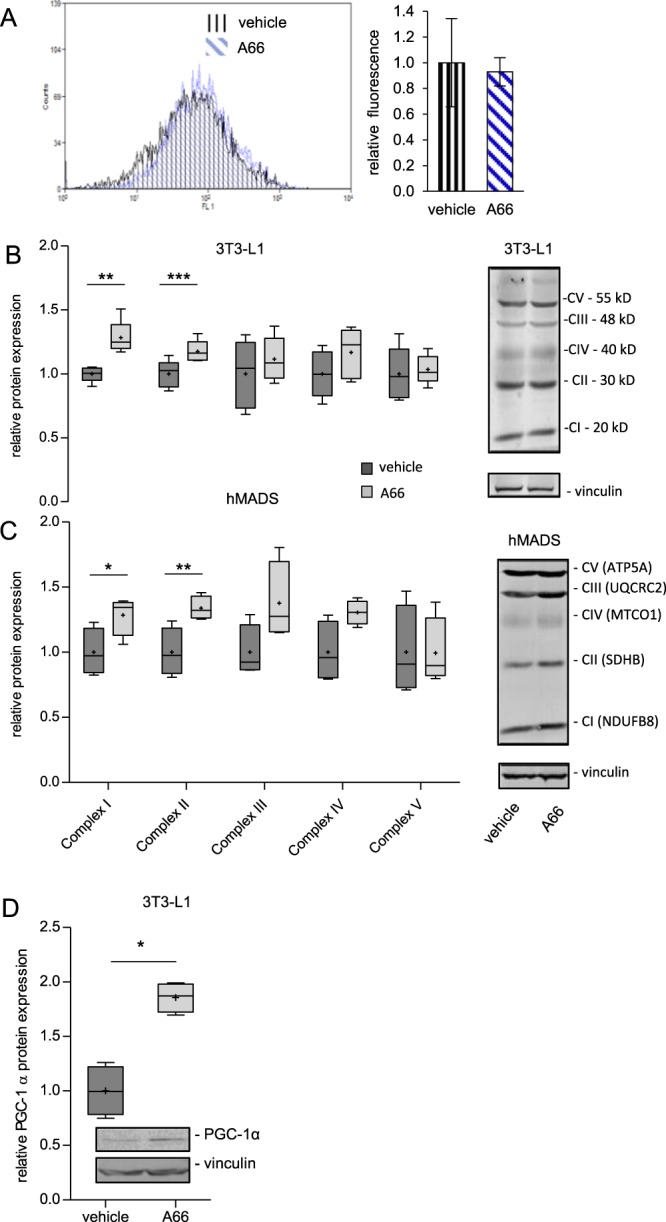
Inhibition of PI3K p110α increases OXPHOS complexes expression levels in 3T3-L1 and hMADS adipocytes. Mouse 3T3-L1 and human MADS adipocytes were treated with the p110α-specific inhibitor A66 for 16 h. (**A**) Mitochondrial mass in 3T3-L1 adipocytes was estimated by staining with Mitotracker Green and FACS analysis. A representative histogram of fluorescent intensity and pooled data from two independent (n = 2) experiments are shown. (**B**,**C**) Expression levels of OXPHOS complexes (I-V) was determined by quantitative immunoblot analysis. Box and whisker plots of data from five (n = 5, 3T3-L1) and four (n = 4, hMADS) independent experiments. Representative OXPHOS immunoblot images are shown next to each graph. CI: NADH dehydrogenase [ubiquinone] 1 beta subcomplex subunit 8 (NDUFB8); CII: Succinate dehydrogenase complex iron sulfur subunit B (SDHB); CIII: Ubiquinol-cytochrome c reductase core protein 2 (UQCRC2); CIV: Cytochrome c oxidase subunit 1 (MTCO1); CV: ATP synthase F1 subunit alpha (ATP5A). (**D**) Expression of PGC-1α in 3T3-L1 adipocytes was determined by immunoblot analysis. Data from four (n = 4) independent experiments. Error bars in A represent standard error of mean. Whiskers in B, C and D represent minimum to maximum value and plus (+) signs the mean value. Statistical significance was tested by paired two tailed t-test. *p < 0.05, **p < 0.01, ***p < 0.001.

Based on the aforementioned mitochondrial gene upregulation, we next examined the functional consequences of p110α inhibition on mitochondrial function in 3T3-L1 and human mesenchymal adipose tissue-derived stem cell (hMADS) adipocytes. Using a mitochondrial probe (MitoTracker Green), we failed to observe any differences in mitochondrial mass between vehicle- and A66-treated cells (Fig. 3A). However, we found that treatment of adipocytes with A66 increased protein expression levels of a number of mitochondrial complex subunits, notably of subunits of NADH dehydrogenase and succinate dehydrogenase, which are components of mitochondrial oxidative phosphorylation complex I and II, respectively (Fig. 3B,C). Also, we found that treatment of 3T3-L1 adipocytes with A66 upregulated protein expression of PGC-1α, a transcriptional co-activator known to promote mitochondrial biogenesis and activity (Fig. 3D). These data are similar to those previously reported for mice subjected to dietary restriction, a well-established life extending intervention, showing that dietary restriction promotes mitochondrial respiration in skeletal muscle by increasing expression of PGC-1α as well as of specific electron transport chain components rather than by increasing total mitochondrial biogenesis^27^.

### Inhibition of the PI3K p110α signalling pathway promotes fat mobilisation

**Figure 4:**
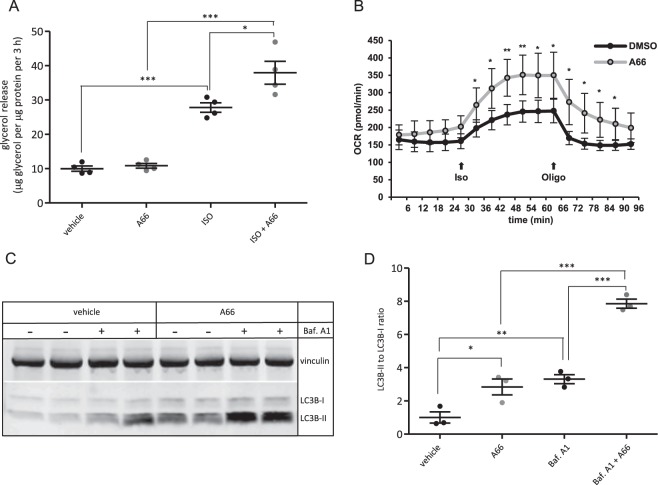
Inhibition of p110α in mouse 3T3-L1 adipocytes promotes fat mobilisation. (**A**) Lipolysis measured as glycerol release from 3T3-L1 adipocytes pre-treated with the p110α-selective inhibitor A66 (1 μM) for 1 h and stimulated with isoproterenol (5 nM) for 3 hours. A scatter plot of data from four (n = 4) independent experiments presented as mean±sem is shown. (**B**) Basal and isoproterenol-stimulated oxygen consumption rate (OCR) in 3T3-L1 adipocytes treated with 1 μM p110α-selective inhibitor, A66. Iso and oligo indicate injection of isoproterenol (1 μM) and oligomycin (1 μg/ml), respectively. Data from a representative experiment performed in quadruplicate are shown as mean±sd. (**C**) Autophagic flux in 3T3-L1 adipocytes treated with 1 μM p110α-selective inhibitor (A66) in the presence or absence of 50 nM Bafilomycin A1 (Baf. A1) assessed by immunoblot detection of LC3B lipidation. Vinculin was probed as a loading control. A representative immunoblot is shown in (**C**) and a scatter plot of data from three (n = 3) independent experiments presented as mean±sem is shown in (**D**). Statistical significance was tested by one way ANOVA with Bonferroni’s multiple comparisons test (A, D) or paired two tailed t-test (B). *p < 0.05; **p < 0.01; ***p < 0.001.

To assess whether inhibition of PI3K also promotes fat mobilisation for oxidation in mitochondria, we also tested the effect of PI3K p110α inhibition on fat metabolism in mouse 3T3-L1 adipocytes. We found that inhibition of p110α by A66 enhanced the rate of lipolysis upon stimulation with the β-adrenergic receptor agonist isoproterenol (Fig. 4A). Moreover, A66-treated adipocytes had a higher level of both basal and isoproterenol-stimulated oxygen consumption (Fig. 4B) consistent with enhanced mitochondrial oxidation. Furthermore, inhibition of p110α increased autophagic flux which has been associated with lipid catabolism through the process of lipophagy^28^, providing further evidence of enhanced lipid catabolism as a result of inactivation of p110α (Fig. 4C,D). Thus, inhibition of p110α in adipocytes stimulates fat catabolic processes through increased lipolysis and mitochondrial oxidation.

Overall, the functional data from the adipocyte models were in line with mitochondrial activity upregulation as an effect of PI3K inactivation, consistent with the gene expression analysis in the fat body of the flies. Taken altogether, these data suggest that upregulation of mitochondrial metabolism might be an important mechanism of life extending effects of inactivation of IIS in model organisms.

## Discussion

The molecular mechanisms underlying the life- and health-span extending effects of loss-of-function mutations in components of the IIS pathway in model organisms remain incompletely defined. Over the recent years, intense efforts have been made to unravel the molecular mechanisms underlying these beneficial effects of IIS downregulation. The IIS pathway plays an essential role in the regulation of lipid and glucose homeostasis. PI3K is a key effector of metabolic actions of the IIS pathway. In the present study, we sought to identify similarities in metabolic effects of PI3K inactivation between fly fat body and mammalian adipocytes. To this end, we have employed transcriptome analysis in both fly fat bodies and mouse adipocytes and assays of fat mobilisation and mitochondrial activity in mouse adipocytes. Transcriptome analysis revealed that inactivation of PI3K resulted in upregulation of genes related to mitochondrial metabolic processes in both fly fat body and mouse adipocytes. Consistent with this, functional assays showed that inhibition of p110α promoted lipοlysis and enhanced mitochondrial activity in mouse adipocytes. These data are consistent with those of a previously reported proteomic study, which demonstrated upregulation of mitochondrial electron transport chain subunits and increased mitochondrial respiration in the fat body of flies with reduced insulin signalling^25^. Taken altogether, such findings lend further support to the notion that increased mitochondrial activity might be part of a conserved mechanism contributing to the lifespan extending effect of IIS pathway downregulation in the adipose tissue.

Interestingly, we found that one of the most prominent gene categories downregulated in Dp110^DN^ overexpressing fat bodies were genes involved in cell cycle regulation. This is consistent with the well-established role of PI3K signalling in promoting cell proliferation, hence the high frequency of PI3K p110α mutation in cancer^29^. However, its potential significance in a postmitotic tissue as the fly fat body remains unknown. The fat body is also a key organ for innate immunity in flies as it produces antimicrobial peptides that combat infection from pathogenic microorganisms^30^. Remarkably, overexpression of Dp110^DN^ in the fat body reduced the expression of genes related to antimicrobial defence. Indeed, amongst the top 100 downregulated genes in *Dp110*^*DN*^ fly fat bodies are several genes encoding antimicrobial peptides and other innate immunity mediators including CecA1, CecA2, AttD and edin (Supplementary Fig. 2). The effect of Dp110^DN^ induction on antimicrobial peptide expression is in line with previous reports implicating the IIS pathway in the regulation of antimicrobial immunity in flies^31,32^.

In line with what is reported here in mouse 3T3-L1 adipocytes, parallel studies in our lab have revealed that genetic or pharmacological inactivation of p110α in the adipose tissue of mice results in enhanced adrenergic signalling, which promotes lipolysis and energy expenditure^19^. The enhancement in adrenergic signalling mitigates the effect of insulin resistance ensuing from p110α inactivation thus producing overall a lean phenotype and improved glucose homeostasis. Lipolysis in the fly fat body is controlled by the adipokinetic hormone (AKH) as well as by the monoamines octopamine and tyramine, the fly functional analogs of catecholamines. Consistent with a role of lipid metabolism in regulation of lifespan, inactivation of the Adipokinetic Hormone Receptor (AKHR) has been shown to result in obesity and reduce lifespan in flies^7^. Similarly, inhibition of octopamine production has been shown to increase fat storage and reduce lifespan in flies^33^. Whether inactivation of Dp110 in the fat body can also potentiate lipolysis and energy expenditure in flies as in mice remains to be examined.

Mitochondrial metabolism upregulation is a reported effect of various interventions shown to extend lifespan. Notably, adipose tissue specific deletion of the insulin receptor results in upregulation of mitochondrial activity^14^. Also, other interventions that increase lifespan, such as calorie restriction and growth hormone receptor knock-out (GHRKO), result in increased mitochondrial activity and/or PGC-1α expression in muscle and liver, respectively^27^. Moreover, insulin being an anabolic hormone that promotes energy storage, has been shown to inhibit the activity of the transcriptional coactivator PGC-1α as well as to suppress mitochondrial biogenesis in hepatocytes^34^. However, the contribution of the specific pathways and effector molecules activated downstream the insulin receptor to these particular phenotypes has not been investigated. This is an important question given that the insulin receptor also activates the Ras/ERK pathway in addition to the PI3K/Akt pathway. Inactivation of the Ras/ERK pathway has also been shown to extend lifespan in flies^35^, though beneficial metabolic effects have so far been reported only in mice^36^. One of the conclusions of the present study is that the reported effects of IIS pathway inhibition on mitochondrial activity can be recapitulated by inhibition of PI3K. The present study identifies PI3K p110α as a mediator of these phenotypes and shows that its inhibition promotes fat mobilisation and mitochondrial metabolism in mammalian adipocytes.

Consistent with upregulated mitochondrial function as result of p110α inhibition reported here, a recent study from our lab has demonstrated reduced age-dependent fat accumulation in mice with adipose tissue-specific p110α deletion using the adiponectin-Cre strain^19^. However, a previous study which used an aP2-Cre strain to delete p110α in the adipose tissue of mice has reported obesity and decreased energy expenditure^37^, which are diametrically different phenotypes from those reported in our study. As discussed in our respective article, there are numerous reports in the literature demonstrating a profound lack of tissue specificity for the aP2-Cre strain, which complicates interpretation of phenotypes. Importantly, the phenotypes described in our previous and present work are consistent with those reported for long term pharmacological inhibition of p110α^20,21^, which adds additional weight to the validity of our findings.

Overall, the present study provides further insights into the mechanistic basis of the beneficial effects of IIS pathway downregulation and highlights conserved biological effects of inhibition of the PI3K/Akt branch of this pathway. As the PI3K/Akt signalling pathway is readily amenable to pharmacological intervention, such information could facilitate translational efforts to produce therapeutics to combat ageing and age-associated metabolic pathologies.

## Methods

### Fly stocks and husbandry

The wild-type stock Dahomey was collected in 1970 in Dahomey (now Benin) and has since been maintained in large population cages with overlapping generations on a 12 h light: 12 h dark cycle at 25 °C. The white Dahomey (*wDah*) stock was derived by incorporation of the *w*^1118^ mutation into the outbred Dahomey background by backcrossing. All stocks were maintained and all experiments were conducted at 25 °C on a 12 h light: 12 h dark cycle at 60% humidity using standard sugar/yeast/agar (SYA) media. For all experiments, flies were reared at standardised larval density, and eclosing adults were collected over a 12 h period. Flies were allowed to mate for 48 h before sorting into single sexes at 10 flies per vial. The dominant negative *UAS-Dp*1*10*^*DN*^ (D954A) (*y*^*1*^w*; P{Dp110D954A}2) (stock number 25918) was obtained from the Bloomington Stock Centre. This stock expresses myc-tagged Dp110 with a defective ATP binding domain under UAS control on the second chromosome^23^. The constitutive fat body driver (*FB-Gal4*) was kindly provided by Sebastian Grönke (MPI for Biology of Ageing, Cologne). Both were backcrossed at least 6 times into the white Dahomey (wDah, *Wolbachia* positive) background.

### Adipocyte culture and differentiation

3T3-L1 mouse pre-adipocytes were grown in DMEM with 4.5 g/L glucose (Gibco, ThermoFisher Scientific) supplemented with 10% Calf Serum (Gibco, ThermoFisher Scientific) and penicillin/streptomycin at 37 °C in a humidified incubator with 5% CO_2_. Cells were allowed to reach confluence. 48 h post-confluence (day 0) cells were transferred in differentiation induction medium consisting of DMEM supplemented with 10% fetal bovine serum (Gibco, ThermoFisher Scientific), 0.5 mM isobutylmethylxanthine (Sigma), 1 μM dexamethasone (Sigma), 1 μM rosiglitazone (Cayman Chemical) and 1 μg/ml bovine insulin (Sigma). 48 h after induction of differentiation (day 2), the medium was replaced with DMEM supplemented with 10% FBS and 1 μg/ml insulin. On day 4, the medium was replaced with DMEM supplemented with 10% FBS. Complete differentiation was normally reached by day 8. hMADS cells were maintained and differentiated to adipocytes as described before^38^.

### Quantitative RT-PCR

Total RNA was extracted from ten whole adult flies per genotype using standard Trizol (Invitrogen) protocols. cDNA was prepared using SuperScript VILO cDNA synthesis kit (Invitrogen) according to the manufacturer’s protocol. Quantitative RT-PCR was performed using the Applied Biosystems 7900HT Fast Real-Time PCR detection system and Power SYBR Green PCR Master Mix (Applied Biosystems). Relative quantities of Dp110 transcripts were determined using the relative standard curve method and normalized to RP49. Primer sequences were:

Dp110-forward: GCAAGATGCGACCGCTATG; Dp110-reverse: GAAACGCGATGGTATGGACT

RP49-forward: ATGACCATCCGCCCAGCATACAGG; RP49-reverse: ATCTCGCCGCAGTAAACG

### RNA-sequencing and bioinformatic analysis

For fly fat body RNA-seq, RNA was extracted using Trizol (Invitrogen) from three biological repeats of twenty five fat bodies dissected from adult females of the following genotypes: *FB-Gal4* and *FB-Gal4* > *Dp110*^*DN*^. All the batching was done so that the treatments to be compared were carried out in parallel. RNA was purified with RNeasy columns (Qiagen) and its quality and concentration were determined using an Agilent Bioanalyzer 2100 (Agilent Technologies, CA, USA). RNA-sequencing was performed on the Illumina platform (paired-end reads of 100 bp sequence) by the Centre for Applied Genomics, Hospital for Sick Children, Toronto, Canada.

For 3T3-L1 adipocyte RNA-seq, adipocytes cultured in 6-well dishes were treated with either 1 μM A66 (Tocris) or vehicle (DMSO) for 24 hours. RNA was extracted using Trizol (Invitrogen) from two biological repeats. RNA was purified with RNeasy columns (Qiagen) and its quality and concentration were determined using an Agilent Bioanalyzer 2100 (Agilent Technologies, CA, USA). RNA-sequencing was performed on the Illumina platform (paired-end reads of 100 base pair sequence) by the University of Utah Genomics Core facility, Salt Lake City, Utah, US.

Tophat (v2.0.11)^39^ was used for alignment to the genome (fly- BDGP5.70, mouse - GRCm38) and HTSeq^40^ to produce transcript counts. DESeq2^41^ was used to determine differential expression. Analysis of the fly data included batch correction based on the day the fat body samples were processed (paired mutant and wild-type samples). Enrichment of functional annotation was performed with gProfiler in R with default correction for multiple testing (g:SCS method)^42^.

### Quantitative immunoblot analysis

Fully differentiated adipocyte monolayers were rinsed with ice-cold PBS and homogenised in a lysis buffer containing 50 mM Tris-HCl pH 7.4, 100 mM NaCl, 50 mM NaF, 5 mM EDTA, 2 mM EGTA, 40 mM beta-glycerophosphate, 10 mM sodium pyrophosphate, 1% Triton X-100 and protease inhibitors. Cell lysates were clarified by centrifugation at 14,000 rpm for 10 min at 4 °C. The clarified supernatants were assayed for protein content using the BCA method according to the manufacturer’s (ThermoFisher Scientific) instructions. 100 µg of total protein were separated by SDS-PAGE and then transferred onto PVDF membranes. 5% non-fat milk was used to saturate the membrane prior to incubation with primary antibodies: murine OXPHOS antibody cocktail (Abcam, cat. no. ab110413); PGC-1a (Cell Signaling Technology, cat. no. 2178); LC3B (Cell Signaling Technology, cat. no. 2775). Signal detection was performed with fluorescently labelled secondary antibodies [anti-mouse IRDye 800-conjugated (Rockland) and anti-rabbit AlexaFluor 680-conjugated (Invitrogen)], using an Odyssey CLx infrared scanner (LICOR). Detection and quantification were performed using the manufacturer’s (LICOR) software (ImageStudio).

### Lipolysis assay

Differentiated 3T3-L1 adipocytes in 12-well dishes, were serum deprived for 16 h in DMEM supplemented with 0.5% BSA. Cells were then washed twice with PBS and incubated in Krebs-Ringer Bicarbonate buffer (30 mM HEPES-NaOH pH 7.4, 10 mM NaHCO_3_, 120 mM NaCl, 4 mM KH_2_PO_4_, 1 mM MgSO_4_, 0.75 mM CaCl_2_) supplemented with 10 mM glucose and 0.5% BSA. Cells were incubated in the presence or absence of 5 nM isoproterenol (Sigma) for 3 h at 37 °C. Glycerol content of conditioned media was determined with Free Glycerol Reagent (Sigma) and normalised to the total protein content of corresponding cell lysates, as determined with the BCA assay kit (ThermoFisher Scientific).

### Measurement of cellular respiration

Mouse 3T3-L1 pre-adipocytes were seeded into 24-well XF24 plates (Seahorse Bioscience) at 5,000 cells per well in 500 μl growth medium. Cells were differentiated to adipocytes as described above. On the day of the experiment, the differentiated adipocytes were washed once with 1 ml of XF Assay Medium Modified DMEM (Seahorse Bioscience) supplemented with 25 mM glucose and 1 mM pyruvate (pH 7.4) and 500 μl of this medium were added per each well. Cellular oxygen consumption rate (OCR) and extracellular acidification rate (ECAR) were measured using a Seahorse Extracellular Flux Analyser (model XF24, Seahorse Bioscience). Compounds were injected at a final concentration of 1 μM Isoproterenol (Sigma) and 1 μg/ml Oligomycin (Cell Signaling Technology). For p110α inhibition, cells were treated with 1 μM of the p110α- selective inhibitor A66 (Tocris) for 3 h before initiation of measurements.

### Mitochondrial content determination

Differentiated adipocytes cultured in 6 well dishes were trypsinised, washed and suspended in 1 ml of cell culture medium containing 100 nM Mitotracker Green (Molecular Probes) and incubated for 30 min at 37 °C protected from light. Stained cells were washed and analysed for green fluorescence emission using a Beckman Coulter Cell Lab QUANTA Flow Cytometer. Mean Fluorescence intensity was calculated using Summit v4.0 software (Dako).

### Statistical analysis

Statistical analysis was performed using GraphPad Prism 5. Specific tests used for analysis of each dataset are described in the respective figure legends.

## Supplementary information

Supplementary Information.

Dataset 1.

Dataset 2.

## Data Availability

The gene expression datasets generated and analysed in this study have been deposited at the NCBI Gene Expression Omnibus. GEO Accession numbers: GSE129430 (Fly fat body data) and GSE129442 (Mouse 3T3-L1 cell data). All other datasets are available from the corresponding author on reasonable request.
