# Supplementary material for: Increased mitochondrial and lipid metabolism is a conserved effect of Insulin/PI3K pathway downregulation in adipose tissue
Source: Sci Rep. 2020 Feb 25;10:3418. doi: 10.1038/s41598-020-60210-3 (PMC7042323; doi:10.1038/s41598-020-60210-3)

Increased mitochondrial and lipid metabolism is a conserved effect of Insulin/PI3K pathway downregulation in adipose tissue

Lucia Bettedi, Anqi Yan, Eugene Schuster, Nazif Alic and Lazaros C. Foukas

Supplementary Information

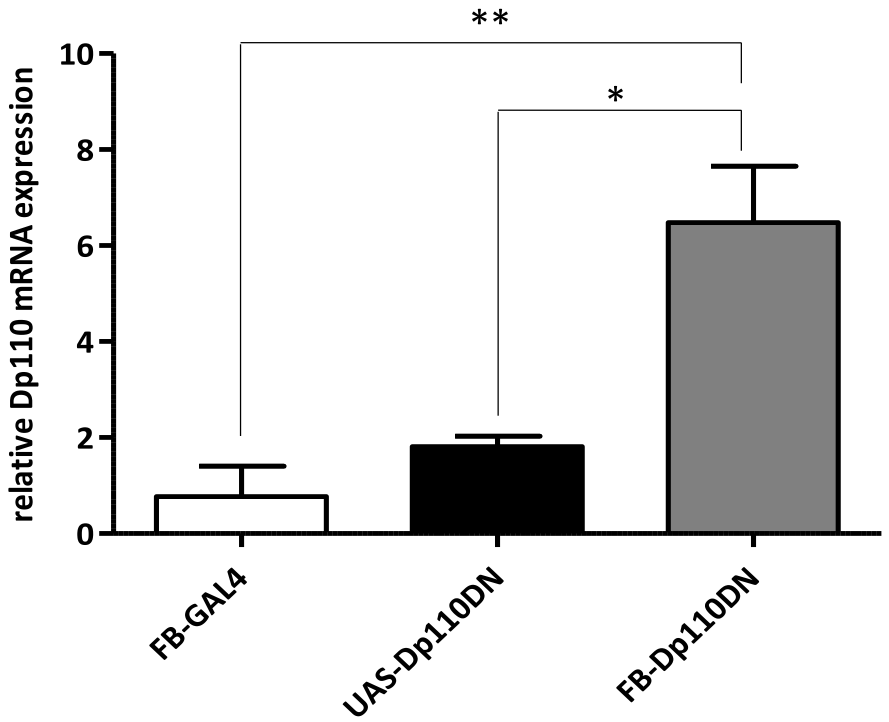

Supplementary Fig. 1: Expression of Dp110DN transgene in fat bodies of FB-Dp110DN flies.

RNA was extracted from freshly dissected fat body homogenates of Dp110DN and control flies and expression of Dp110 was assessed by Q-PCR.

Data are presented as mean±sem from three (n=3) biological replicates (each sample derived from 5 pooled fat bodies). Statistical comparison was performed with one way ANOVA with Bonferroni's multiple correction test. * $p<0.05$, ** $p<0.01$.

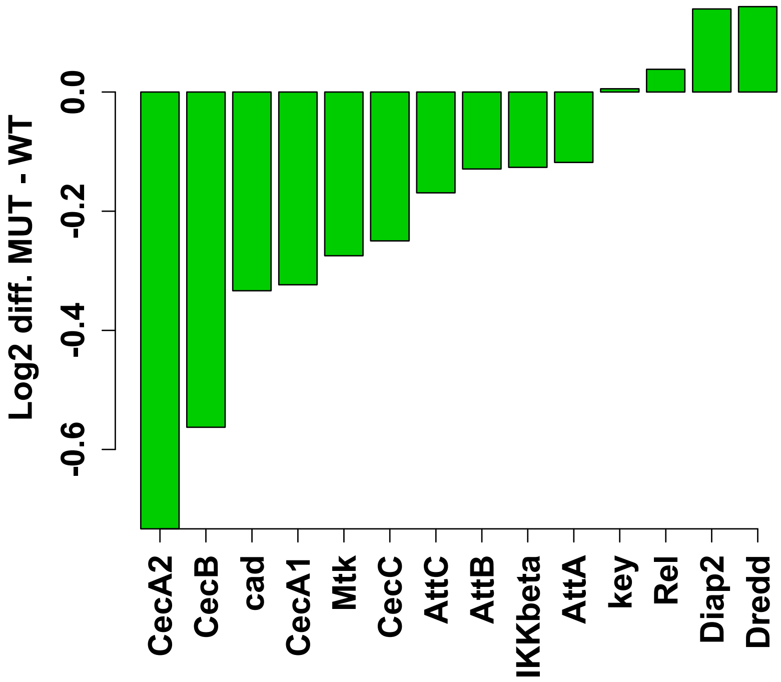

Supplementary Fig. 2: Reduced antimicrobial peptide gene expression in Dp110-DN fly fat body.
Antimicrobial humoral response (GO:0019730) shows a significant shift for down regulation in the mutant flies (p = 0.004, Mann-Whitney test on log2 diff).

Source blots for Fig. 3B-C

3T3-L1

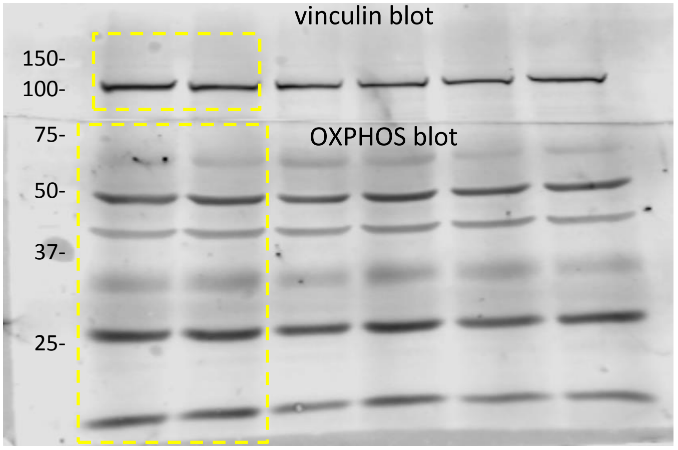

hMADS

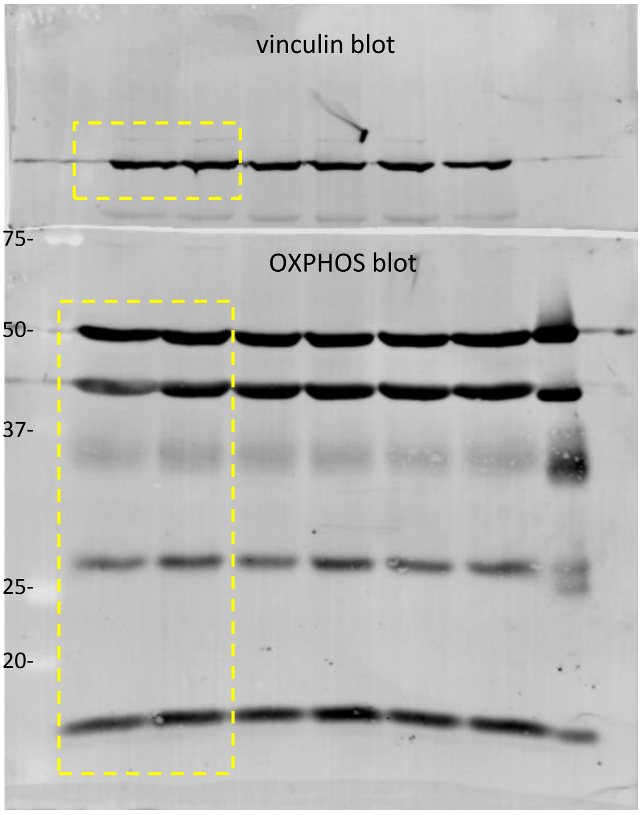

Source blots for Fig. 3D

PGC1α blot

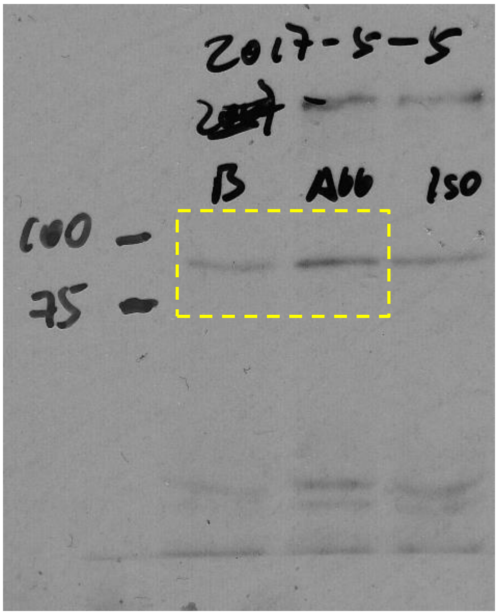

vinculin blot

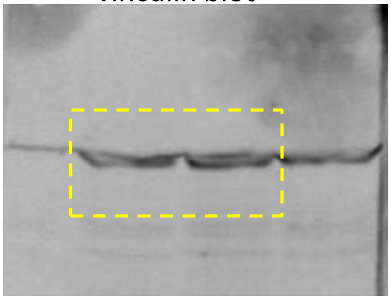

Source blots for Fig. 4C

vinculin blot

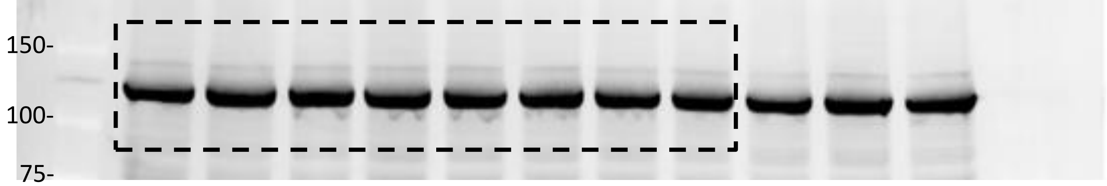

LC3B blot

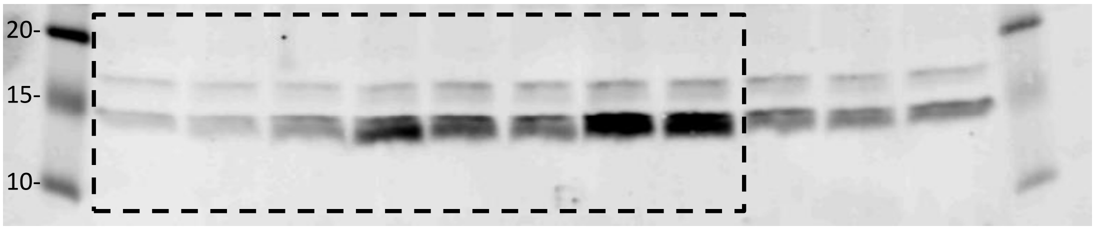

Supplement: Supplementary file 1 — Supplementary Information. [file 41598_2020_60210_MOESM1_ESM.pdf]
